# Factors Influencing Successful Breastfeeding: A Regional Survey and Comparative Review in European and Global Contexts

**DOI:** 10.3390/children13081078

**Published:** 2026-08-14

**Authors:** Pasqua Anna Quitadamo, Matteo Rinaldi, Simona Pesce, Laura Comegna, Michele Bisceglia, Gianfranco Maffei, Luigi Corvaglia, Mariella Elisabetta Baldassarre

**Affiliations:** 1 NICU, Casa Sollievo Della Sofferenza-IRCCS, l’Ospedale Fondato da Padre Pio, 71013 San Giovanni Rotondo, Italy; 2 Neonatology and Intensive Care Unit—Neonatal Ward and Neonatal Emergency Transport Service, Policlinico Foggia Hospital, 71122 Foggia, Italy; 3 UOC Neonatologia e TIN, AOR San Carlo Potenza, 85100 Potenza, Italy; 4 Neonatology and Neonatal Intensive Care Unit, S.Orsola-Malpighi Hospital, 40138 Bologna, Italy; 5 Department of Interdisciplinary Medicine, Neonatology and NICU, Aldo Moro University, 70121 Bari, Italy; mariaelisabetta.baldassarre@uniba.it

**Keywords:** breastfeeding promotion, skin to skin, rooming in, neonatal care, cross-sectional survey

## Abstract

**Background/Objectives**: Breastfeeding is universally recognized as essential for neonatal health, offering benefits that extend into later life stages. The World Health Organization (WHO) advocates for increasing access to effective, low-cost interventions that promote successful breastfeeding, such as skin-to-skin contact (SSC), kangaroo mother care (KMC), and rooming-in. This study aimed to assess the local context regarding the use and dissemination of practices such as prenatal classes, immediate and post-delivery SSC, rooming-in, and the management of discharge and post-discharge care. **Methods:** This study is a cross-sectional survey across all 29 birth centers in two regions of Southern Italy. An online questionnaire was distributed to all neonatal units, and local data were compared with national, European, and global datasets. **Results:** Childbirth preparation classes were offered by 38% of centers. Fifteen centers routinely implemented SSC in the first two hours postpartum, while 12 centers used SSC for a shorter duration. Rooming-in was practiced in all centers, with 8 of 29 centers (27.6%) offering it on a full 24 h basis. A brief postpartum follow-up dedicated to breastfeeding support was provided in 12 of 29 centers (41.4%), and 6 centers offered a dedicated telephone breastfeeding consultation service. **Conclusions:** This survey confirmed the value of monitoring adherence to breastfeeding promotion practices at the regional level, within a broader national and global framework. Differences between units indicate the need for targeted interventions to implement all known, evidence-based methods for promoting breastfeeding initiation.

## 1. Introduction

The United Nations Convention on the Rights of the Child, adopted by the General Assembly in 1989 [1] and incorporated into Italian law [2], affirms that children have the right to care without separation from their parents (Article 9) and to the highest attainable standard of health (Article 24). A strong start in life is a fundamental component of the global strategy for the health of women, children, and adolescents (2016–2030) and aligns with the 2023 World Health Organization resolution aimed at accelerating progress in maternal, neonatal, and child survival. WHO advocates for expanding access to proven, high-impact interventions such as breastfeeding, kangaroo mother care, and parental closeness, these being cost-effective strategies with exceptionally high returns [3].

Regarding the perinatal period, the global health community has issued specific recommendations on essential newborn care (ENC) [4], encompassing interventions during the initial hours and days of life, an especially critical window where timely actions markedly influence health outcomes. ENC components include thermal regulation, early and exclusive breastfeeding, and prompt monitoring and treatment of low birth weight or ill neonates. The overarching goal is to enable countries to safeguard infant health through simple, life-saving measures.

Breastfeeding confers substantial health benefits and survival advantages for neonates. Effective promotion and support of breastfeeding necessitate facilitating practices such as immediate skin-to-skin contact, early initiation of breastfeeding, rooming-in, and limiting supplementation with formula milk in breastfed infants. These practices are promoted by the Baby-Friendly Hospital Initiative (BFHI) [4,5,6,7]. Despite variable international implementation, progress in Italy has been limited over the past three decades, with fewer than 1% of hospitals accredited as “baby-friendly” [8]. In 2023, the “Hospital Policies on Breastfeeding Project” was launched to promote breastfeeding across Italy independently of BFHI accreditation, as a joint initiative involving national scientific societies specializing in perinatal care.

The WHO (www.who.int/health-topics/breastfeeding) (accessed on 20 Decembre 2024), the Italian Ministry of Health (www.salute.gov.it/portale/allattamento) (accessed on 20 December 2024) [9], and relevant scientific societies recommend exclusive breastfeeding for the first six months of life, continuing alongside complementary feeding, with extension up to two years or beyond based on maternal and infant preferences. Policymakers and healthcare professionals are strongly encouraged to promote optimal breastfeeding practices in both hospital and community settings by adopting organizational policies and clinical protocols grounded in scientific evidence and shared among multidisciplinary teams.

KMC is a well-established, evidence-based, life-saving intervention. SSC, which involves placing a naked neonate prone on the mother’s bare chest immediately after birth, enhances thermal regulation, promotes neonatal relaxation, and fosters mother–infant bonding. SSC is among the most effective facilitators of breastfeeding and should ideally commence as soon as possible, lasting at least one hour [10,11]. Conversely, maternal–neonatal separation has been shown to negatively influence breastfeeding initiation and duration [4,5,6,7,12].

During SSC, the mother and neonate may be gently covered with a pre-warmed blanket or cloth. The neonate should remain in this position until successful initial breastfeeding is established [13]. The first two hours postpartum constitute a critical period for stabilizing breastfeeding and promoting its continuation, as neonatal feeding behaviors, such as rooting and sucking, are highly active during this window, alongside responses to tactile, thermal, and olfactory stimuli from the maternal body.

A 2016 Cochrane review [14] synthesized evidence on the effects of SSC on breastfeeding outcomes. Despite heterogeneity and variable quality between the included studies, SSC was associated with higher breastfeeding efficacy scores and longer durations of both exclusive and total breastfeeding [14]. Evidence suggests a dose-response relationship between the duration of initial SSC and the likelihood of exclusive breastfeeding during the hospital stay [7]. Systematic reviews and meta-analyses have demonstrated positive correlations between skin-to-skin contact within the first hour and successful initiation and sustained breastfeeding, including exclusivity up to 3–6 months [14,15,16,17].

Nevertheless, despite unequivocal evidence supporting the benefits of prolonged SSC, adherence remains suboptimal, and the optimal duration has yet to be definitively established. Although SSC is a simple, natural intervention, some centers have reverted to routine neonatal separation postpartum.

The American Academy of Pediatrics (2012) indicated that routine neonatal assessments and interventions can be performed during skin-to-skin contact or delayed until after it, provided there are no maternal or neonatal complications [17]. Prolonged SSC (>45 min) has been linked to extended breastfeeding duration [18].

Rooming-in, the practice of keeping mother and neonate together during the postpartum hospital stay, is among the most widely adopted maternity care practices globally, including in Italy and other high-income countries. Often implemented in a partial (non-24 h) form, rooming-in plays a key role in promoting breastfeeding initiation [19,20,21], by enabling mothers to respond promptly to infant cues such as hunger, rooting, or suckling. This close contact facilitates increased breast stimulation, enhances the milk let-down reflex, and accelerates lactogenesis II, the physiological process of milk “coming in.” This milestone is crucial for sustained breastfeeding, ensuring adequate infant intake for weight gain and bolstering maternal confidence and satisfaction [22,23,24,25].

Beyond the mechanics of feeding, rooming-in serves as a vital phase of postpartum caregiving. Active participation in neonatal care during the initial days, ideally with partner or caregiver support, helps parents develop practical skills, reduces anxiety during the transition home, and fosters a confident, less stressful postpartum experience [26,27]. Successful implementation of these practices requires a supportive clinical environment, with trained healthcare personnel to supervise, guide, and support both mother and infant. This underscores the importance of institutional policies and professional education. While the facilitating effects of these practices on breastfeeding initiation are well recognized, less is known about adherence levels, which are critical for improving outcomes. Identifying and addressing barriers to adherence, as well as emphasizing factors associated with successful implementation, are essential.

This study aimed to assess the diffusion of key breastfeeding promotion practices, including prenatal education classes, immediate and postpartum skin-to-skin contact, rooming-in, and the management of discharge and post-discharge care. A comparative analysis was conducted between Level I and Level II healthcare facilities to evaluate adherence to these procedures, complemented by statistical assessments to inform targeted interventions. Employing an inductive approach, the findings were contextualized within broader national and international frameworks.

This cross-sectional study, supplemented by a comprehensive literature review, provides an integrated overview of the dissemination of evidence-based breastfeeding promotion practices, initially focusing on Southern Italy and expanding to a global context. Mapping the current state of neonatal care in this domain offers a valuable tool for assessing practice implementation and identifying areas for improvement. The results serve as both a snapshot of current practices and a reproducible framework for ongoing monitoring in other regions and time points. Ultimately, the objective is to enhance breastfeeding initiation and duration rates, thereby improving neonatal health outcomes locally and globally.

## 2. Materials and Methods

A cross-sectional survey was conducted across neonatal units within two regions of Southern Italy, Puglia and Basilicata. The study was carried out in accordance with the STROBE guidelines. All neonatology centers within the territories of these regions were eligible for participation. Each center was contacted directly via email or telephone, reaching the unit director personally, who received a formal participation request from the study coordinators. The director designated a pediatrician/neonatologist to complete the survey. As detailed in the study protocol provided to each participating center, the assigned clinician was responsible for analyzing and collecting relevant data from their respective units before completing the questionnaire.

The neonatal unit directors consented to share this data with other participating centers and the broader scientific community. Data submission was deadline-driven, with a one-month period allocated for completion. Quantitative data concerning the number of births and the types of units pertain to the year 2024, while all other data relate to the implementation and diffusion of practices over the previous three years (2022–2024).

Data collection was carried out by distributing a structured online questionnaire (Annex) to all regional neonatal units. Upon completion, responses were systematically evaluated, integrated into a dedicated database, and subjected to statistical analysis. The comparisons focused on assessing adherence to clinical practices in relation to the type of neonatal unit and the number of births annually.

### 2.1. Setting

The affiliated centers encompass all currently active centers in the regions of Puglia and Basilicata. Out of a total of 29 units, 4 are located in Basilicata, while the remaining 25 are situated in Puglia.

In Puglia, 4 centers are located in the provincial capital, Bari, comprising 2 Level II and 2 Level I facilities. These include 3 hospital trusts, one of which is a university hospital, and 1 accredited private hospital. In the province of Bari, 4 hospitals are located, consisting of 1 Level II and 3 Level I facilities.

Further north in Puglia, 4 hospitals are situated in the province of Foggia, with 2 designated as Level I and 2 as Level II. The province of Barletta-Andria-Trani (BAT) has 3 Level I hospitals. The province of Taranto hosts 3 facilities, including the Taranto hospital, the only Level II facility in the province.

The province of Lecce is represented by 4 hospitals, including the city’s hospital, which is a Level II unit, and 3 Level I facilities. Regarding birth volumes, 19 centers report fewer than 1000 deliveries annually, while 2 centers perform between 1000 and 1500 deliveries per year. Five centers have an annual birth count ranging from 1500 to 2000, and 3 centers report between 2000 and 2500 births per year. These data pertain to the year 2024.

### 2.2. Definitions

Provinces: The provinces of the two regions, Puglia and Basilicata, are as follows:

- Puglia: Bari (regional capital), Foggia, Taranto, Brindisi, BAT (Barletta, Andria, Trani), Lecce
- Basilicata: Matera, Potenza.

Type of Hospitals:

- First Level Hospital (Level I Emergency Department—ED):
  - Functions: Management of emergency-urgent admissions (Level I ED), short-stay observation, resuscitation, general medicine and surgery.
  - Specialties: Includes internal medicine, general surgery, anesthesia, orthopedics, cardiology with coronary care unit (UTIC), neurology, oncology, ophthalmology, otorhinolaryngology, urology.
  - Population Served: Territorial catchment area between 150,000 and 300,000 inhabitants.
- Second Level Hospital (Level II Emergency Department—ED):
  - Functions: Provides all services of Level I plus high-specialization functions such as cardiac surgery, neurosurgery, neonatal intensive care (TIN), vascular surgery, thoracic surgery, and burn centers.
  - Population Served: Territorial catchment area between 600,000 and 1,200,000 inhabitants.
  - Structures: Usually identified as university hospitals, university-affiliated hospitals, or large ASL (Local Health Authority) facilities (an Italian public entity managing health services, prevention, care, rehabilitation, on a territorial basis).

Consulting Services:

- Family clinics are territorial socio-health services within the National Health Service offering free multidisciplinary counseling and assistance. They promote the health of women, couples, minors, and families through psychological, social, gynecological, and obstetric support.

Rooming-in:

- Total: The newborn is cared for in the same room as the mother, including visits and any procedures or 24/24 h except for visits, check-ups, and screening (max 2 h).
- Partial: Visits, check-ups, and screening are performed in the nursery; the mother has the option to leave the newborn in the nursery overnight.

### 2.3. The Questionnaire

This survey was promoted by the regional section of SIN Puglia Basilicata and involved 29 Centers. The proposed questionnaire included first questions to outline the healthcare facility’s structure, followed by questions related to four key moments of the pregnancy/birth pathway: the prenatal period, the moment of birth, the hospital stay, and discharge.

The questionnaire employed a straightforward format, consisting of 14 questions with binary (yes/no) response options. The first questions concerned the type of structure and operational unit, the annual number of births, and whether the hospital held “Baby-Friendly Hospital” status.

The second set of questions focused on the prenatal phase, specifically addressing prenatal information delivery. It inquired whether pregnant women receive guidance through childbirth preparation courses led by midwives, and whether these sessions are held within the hospital or in community settings such as consulting services. It also assessed the percentage of mothers participating in childbirth preparation activities. The final question in this section asked whether the center provides informational materials on postnatal practices, during pre-admission procedures or during childbirth preparation meetings.

The questions concerning the delivery moment focused on SSC. Specifically, centers were asked if SSC was routinely facilitated within the first two hours after birth.

If the answer is affirmative, they are asked whether the mother–infant dyad is monitored during this time and if this practice leads to the postponement of procedures such as weighing, bathing, dressing, and prophylaxis. In the case of a negative answer, they are asked if the possibility of SSC is still offered immediately after birth, albeit for a duration shorter than the recommended two hours.

The next section investigated rooming-in practices. Questions addressed whether rooming-in was implemented and, if so, whether it was total or partial. The survey also assessed whether parents received information, both verbally and/or in written form, about the importance of rooming-in and potential (albeit rare) risks, and whether informed consent was obtained. Furthermore, the questionnaire addressed whether the partner or a trusted caregiver was permitted to be present in the mother’s recovery area for 24 h a day.

The final section of the survey addressed discharge practices. Specifically, it asked when healthy newborns were typically discharged from the hospital, with three options provided: between 48 and 72 h, after 72 h, or another timing.

It then asks if the discharge letter has a pre-defined checkbox for prescribing formula milk, and if a short-term follow-up dedicated to breastfeeding is guaranteed after discharge—with nursing/midwifery staff, and an outpatient visit with medical staff—with three response options (Yes for all, Yes only for the first, No).

### 2.4. Statistical Analysis

Statistical analysis was performed utilizing Jamovi statistical software (Version 2.3; Jamovi project, 2022). Descriptive statistics were applied to summarize the data; specifically, categorical variables were expressed as absolute frequencies and corresponding percentages.

To explore potential relationships between variables and identify statistically significant correlations, Pearson’s or Spearman’s correlation coefficients were computed based on the underlying data distribution (parametric or non-parametric, respectively). Furthermore, to evaluate associations between qualitative (categorical) variables, contingency tables were generated. These associations were subsequently analyzed using the Chi-square test or Fisher’s exact test, as appropriate, depending on expected cell counts.

## 3. Results

### 3.1. Response Rate

The response rate by participants was 100% for 18 of 22 questions. Two questions had two missing responses each, one had a single missing response, and a single question, regarding the postponement of procedures during skin-to-skin contact, had 16 out of 29 missing responses. nine participants did not respond to question related to the postponement of procedures. Another nine participants failed to answer two questions: for eight of them, the missing responses concerned procedural delays and support during SSC, while for one participant, the unanswered questions pertained to procedural delays and the presence of a caregiver. Additionally, one doctor did not answer four questions, which addressed procedural delays, the responsibility for support during SSC, the availability of outpatient medical visits, and the accessibility of telephone consultations (Table 1). We do not know the reasons for the lack of responses.

### 3.2. Prenatal Section

**Figure 1:**
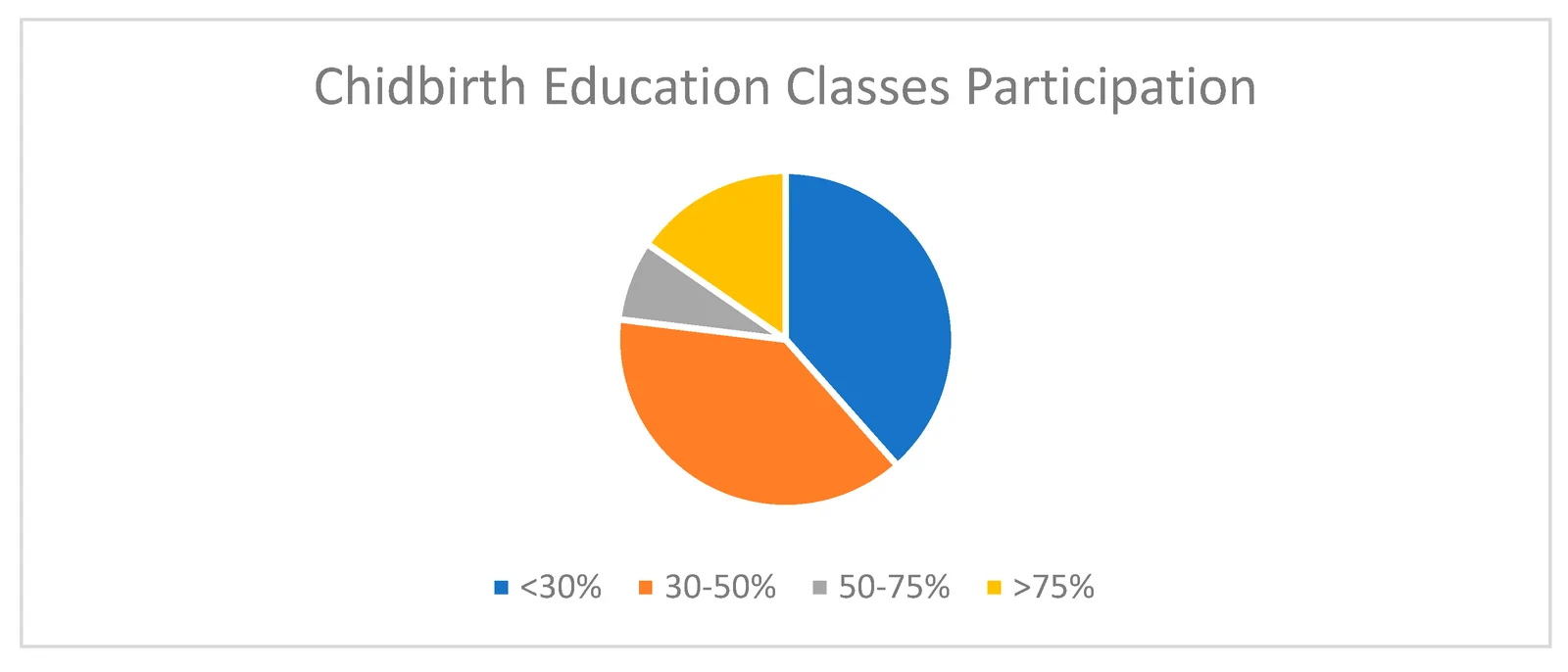
Percentage distribution of participation in childbirth education classes.

**Childbirth Preparation Classes**: 38% (11/29) of centers offer childbirth preparation classes for pregnant women. The proportion of women enrolled in these classes is below 30% in 34.5% (10/29) of centers, between 30% and 50% in another 34.5% (10/29), between 50% and 75% in 6.9% (2/29), and exceeds 75% in 13.7% (4/29) of centers (Table 1, Figure 1).

**Educational Materials**: Approximately half of the centers (51.7%, 15/29) report providing printed informational materials regarding postnatal practices during pregnancy lessons.

### 3.3. Post-Natal Section

**Post-birth Procedures Delay**

**Figure 2:**
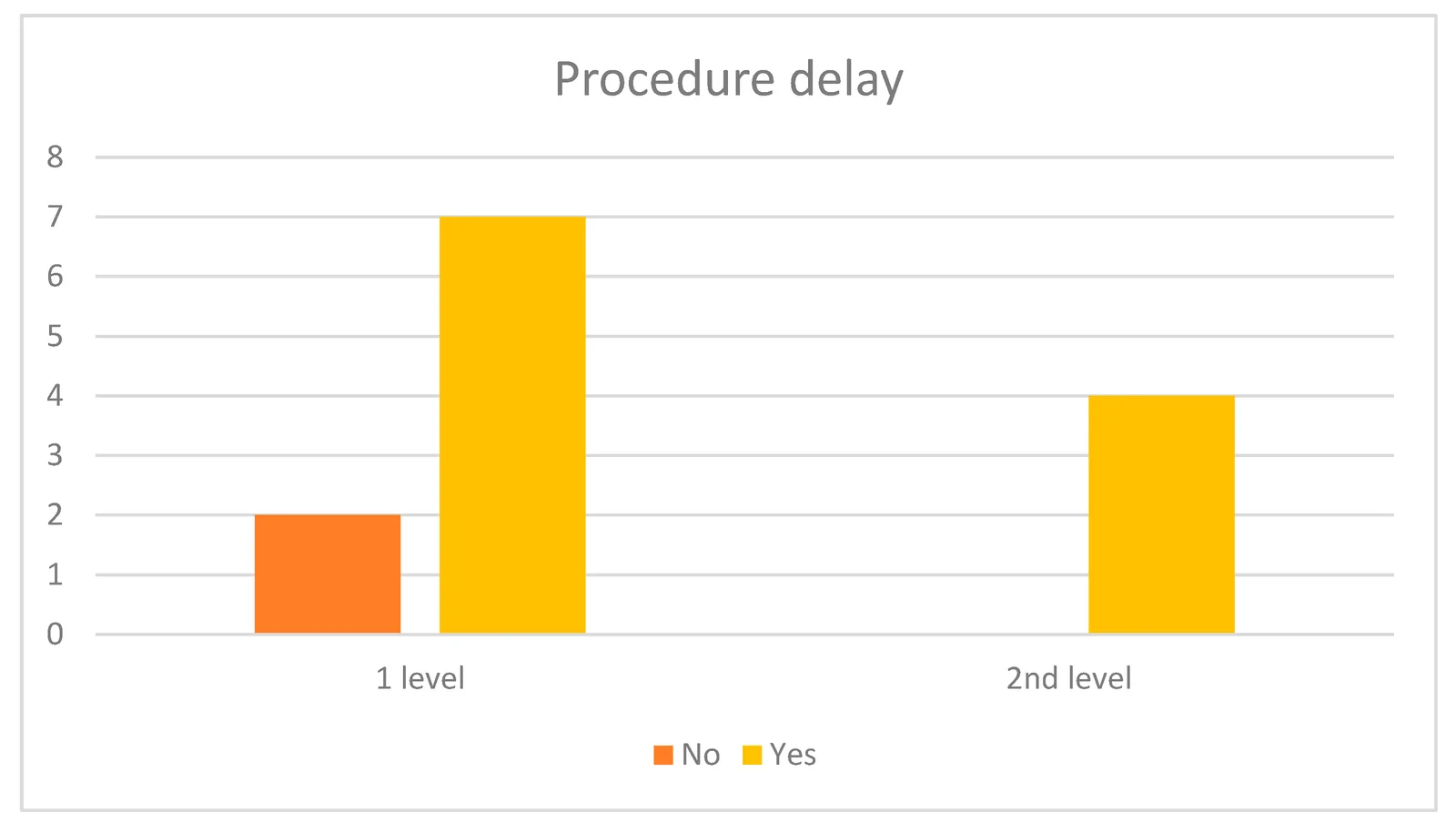
Delay in procedures’ distribution in the first and second level centers.

Post-birth procedures, including weight measurement, bathing, dressing, and prophylactic interventions, are deferred in 11 out of 13 responding centers, whereas these procedures are conducted without delay in the remaining two centers (Figure 2, Table 1 and Table 2). Differences between primary and secondary care centers were not statistically significant (Table 3).

**Figure 3:**
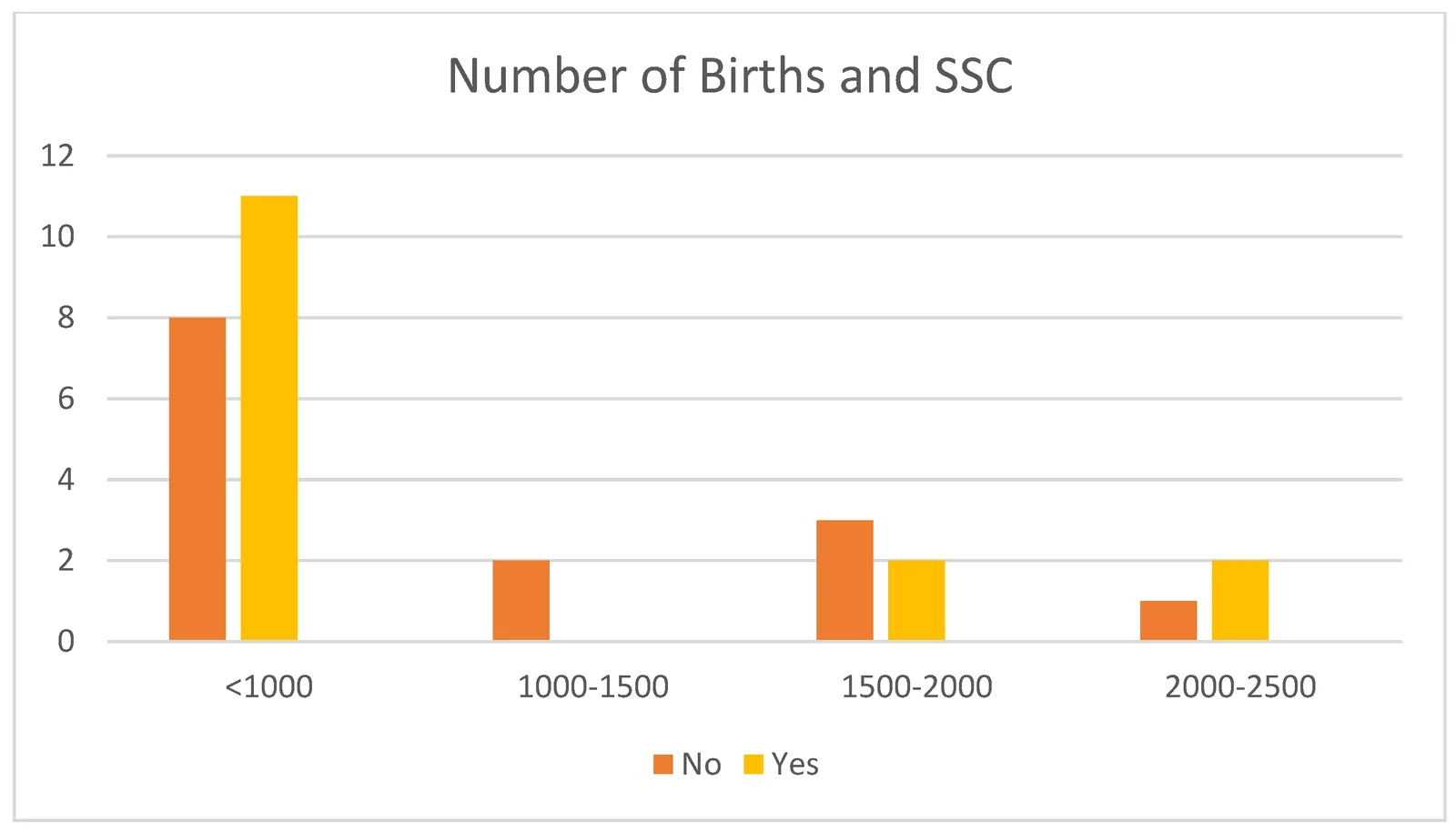
Adherence to skin to skin by n° of births

**SSC**: Routine implementation of SSC within the first two hours postpartum is reported by 15 centers. An additional 12 centers utilize SSC but for durations shorter than two hours. SSC is not practiced after birth in two centers. Monitoring of SSC during the postpartum period is performed routinely by nurses in 15 hospitals, neonatologists in 4 centers, and midwives in 1 center. Data regarding monitoring in the remaining nine centers are unavailable (Table 1 and Table 4). Participation in Level I centers prevails, without statistical significance (Table 5). No statistically significant correlation was found between the type of unit and adherence to skin-to-skin practices within the first two hours after delivery, stratified by n° of births (Figure 3).

### 3.4. Rooming-In

**Figure 4:**
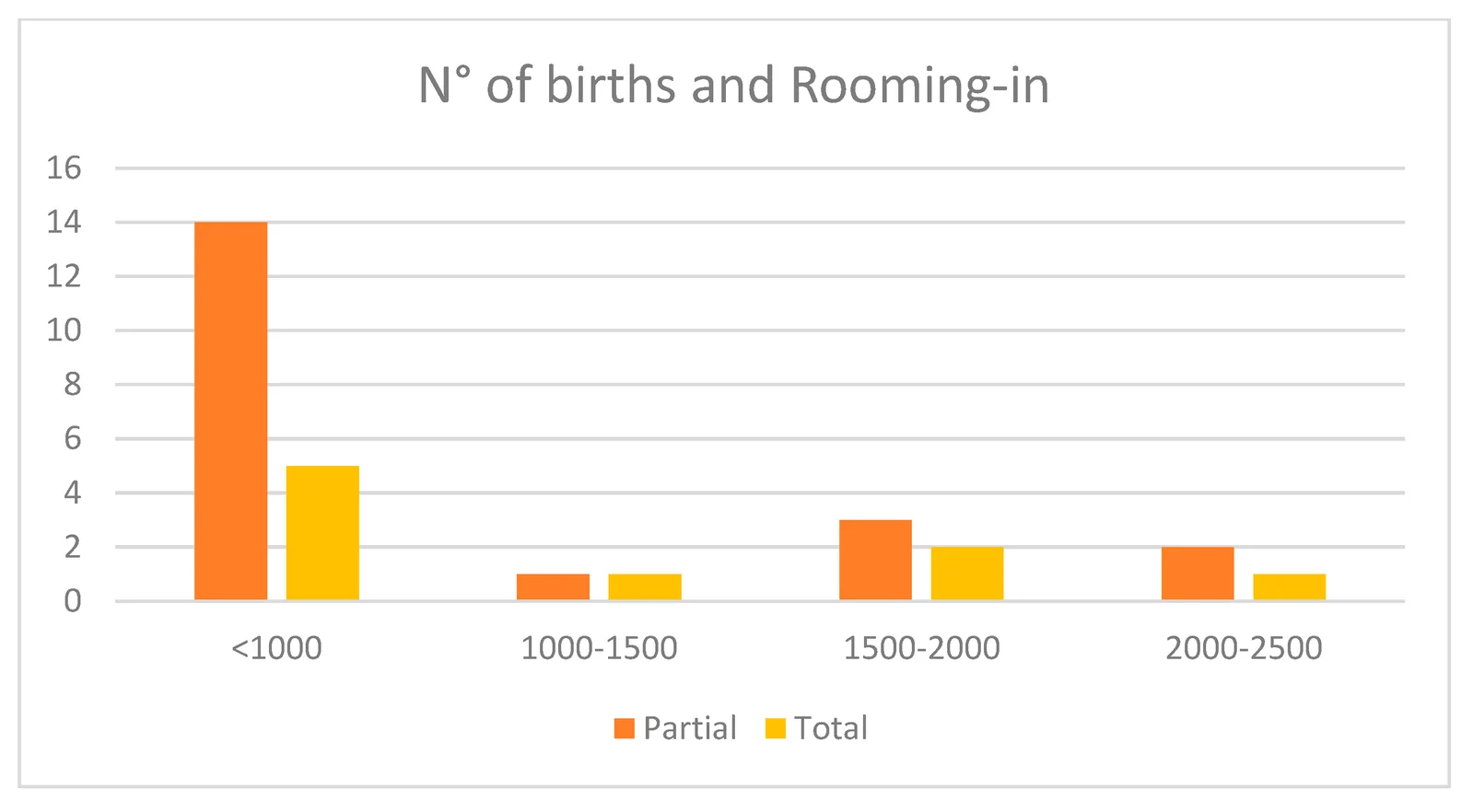
Type of rooming-in and distribution by n° of births.

All surveyed centers (100% response rate) reported practicing rooming-in. Among these, eight centers (27.6%) provided continuous, 24 h rooming-in services. When queried about parental awareness, 21 centers (73%) confirmed that parents are informed, either orally, in writing, or both, regarding the importance of rooming-in and its potential risks. Additionally, written consent for rooming-in is obtained in nine centers (31%). In 14 centers (48.3%), the presence of a caregiver in the same room as the mother post-delivery is permitted (Table 1). In Figure 4, the distribution of rooming types is illustrated in relation to the number of births. Correlation between rooming-in and type of unit (Table 6) was not found statistically significant (Table 7).

### 3.5. Discharge

**Figure 5:**
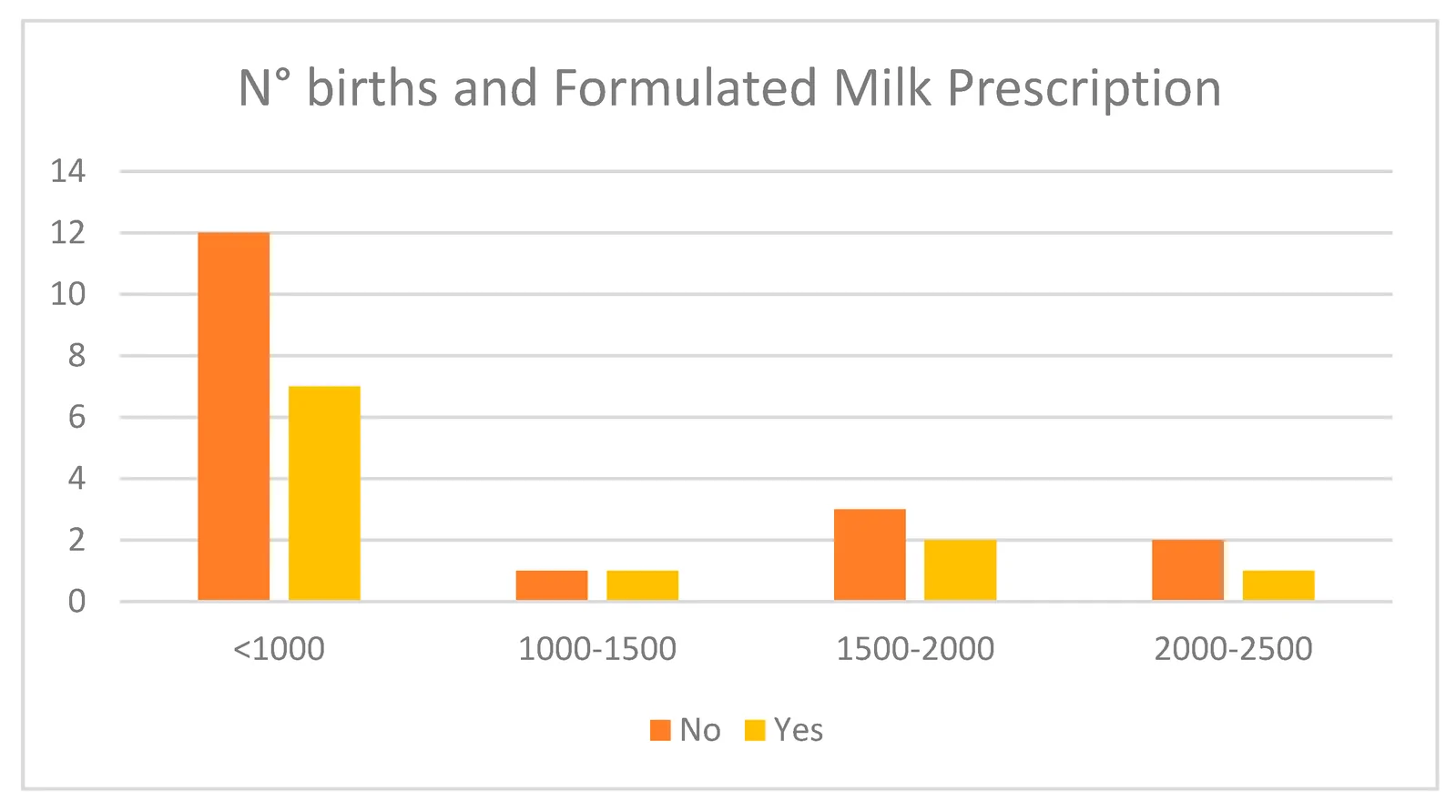
Prescription of formula milk at discharge. Distribution by n° of births.

All centers, except one, discharge patients between 48 and 72 h postpartum; in a single center, the length of hospital stay exceeds 72 h. In 11 hospitals (39.3%), the discharge letter specifies the type of formula milk when a medical indication for its use is present. (Table 1, Figure 5).

### 3.6. Post-Discharge Section

**Figure 6:**
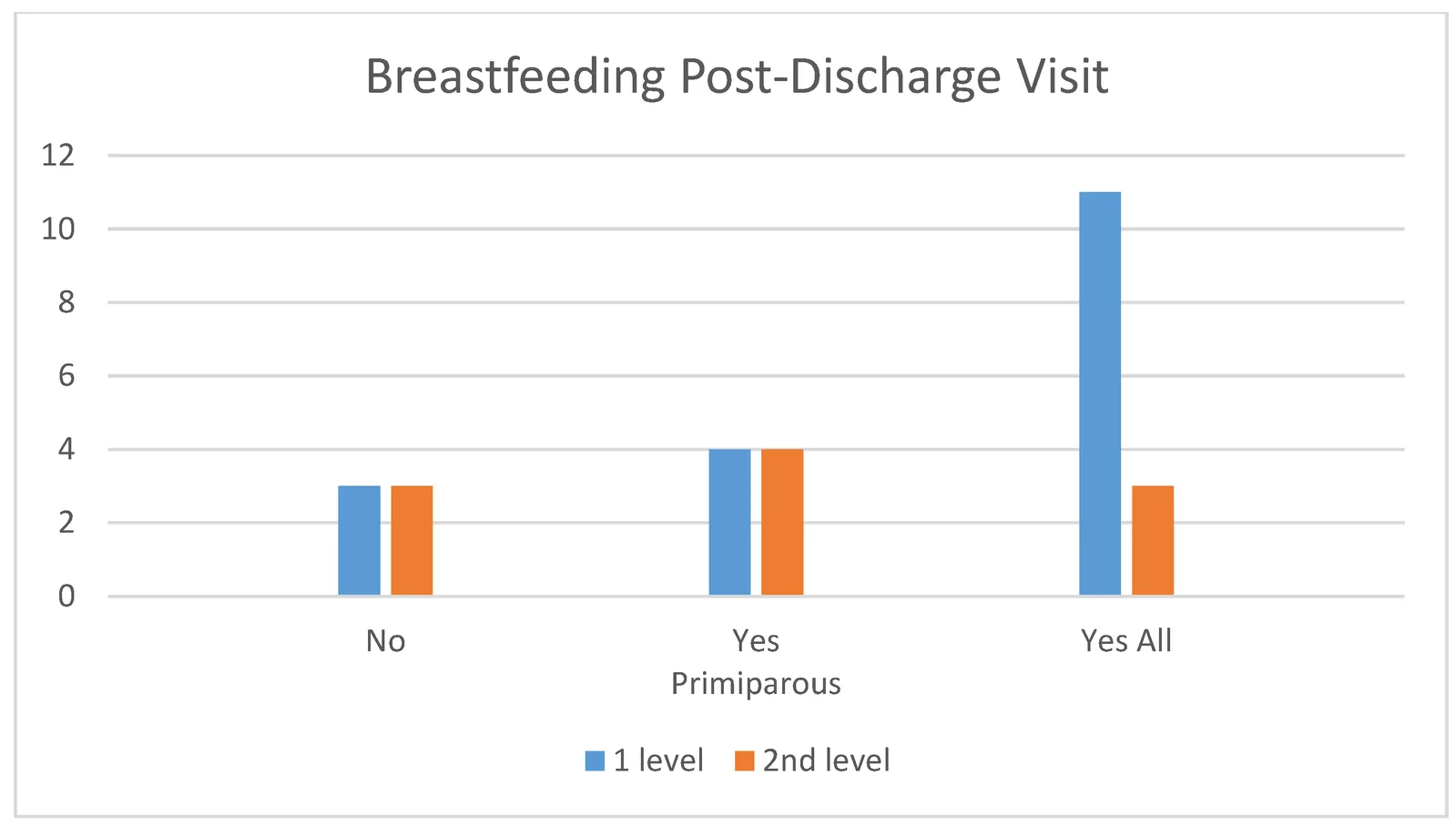
Availability of a post discharge visit dedicated to breastfeeding and type of unit.

Regarding post-discharge support, 12 out of 29 respondents (41.4%) confirmed that a brief follow-up, conducted by nursing or midwifery staff, specifically dedicated to breastfeeding support, is routinely provided. Concerning outpatient visits following discharge, 14 centers (50%) ensure a medical consultation for all newborns within the first week, while 8 centers (28.6%) offer such visits exclusively to primiparous mothers. Six centers do not provide this service, and one response was missing. Six centers offer a dedicated telephone breastfeeding consultation service, whereas 21 centers do not, with 2 centers not responding to this question (Table 1). The distribution of availability of post-discharge breastfeeding support services by unit type is illustrated in Figure 6 and detailed in Table 8.

No statistically significant association was identified between the availability of post-discharge breastfeeding support and the type of healthcare unit (Table 9).

## 4. Discussion

Most interventions aimed at promoting breastfeeding are highly effective, feasible, and cost-efficient across diverse settings. Omitting initiatives with a favorable cost-benefit ratio raises ethical concerns and constitutes a significant medical oversight. We argue that monitoring the implementation of these practices should be a priority for research, as systematic measurement is essential for identifying areas of weakness, addressable through targeted, evidence-based interventions, and for highlighting successful strategies for dissemination.

This survey provides a snapshot of a critical aspect of neonatal health within our region, focusing on the immediate postpartum period—a particularly sensitive window. Gaining insight into this phase is fundamental for informing quality improvement strategies across various stages of the perinatal care pathway and for ultimately achieving breastfeeding targets recommended by international health organizations. Interventions should and can be initiated even prior to birth.

Our analysis, which traces the continuum from gestation to post-discharge, reveals that participation in childbirth preparation courses remains suboptimal: approximately 70% of the population has a participation rate below 50%. Notably, 34% of the cohort exhibits an adherence rate below 30%, and only 50% of healthcare services provide informative materials, in both print and digital formats. Participation in these courses is strongly associated with successful initiation and continuation of breastfeeding, as they serve as vital preparatory opportunities. Well-informed and prepared women approach breastfeeding with greater confidence, are better equipped to manage potential challenges, and demonstrate increased awareness of its importance [28,29,30]. These factors collectively contribute to improved breastfeeding outcomes [31].

Consequently, healthcare policymakers should prioritize expanding access to prenatal education for the pregnant population. Additionally, the provision of informational materials, including digital resources, is essential to enable expectant mothers to access reliable answers to their questions, thereby fostering informed decision-making based on professional guidance rather than unverified sources.

Our commitment included a comparative analysis across three levels, global, European, and national, for each of the assessed indicators. However, we encountered significant challenges due to the lack of precise, up-to-date, and standardized data on participation in prenatal education. Data collection remains fragmented, often derived from disparate national or regional surveys employing heterogeneous methodologies. Most reports from WHO and UNICEF focus predominantly on prenatal care indicators, such as clinical consultations, rather than participation in specific educational programs.

Despite these limitations, emerging evidence suggests considerable variability in participation rates, largely influenced by socioeconomic and healthcare system factors. In high-income countries—such as North America, Western Europe, and Australia, participation in group prenatal education ranges between approximately 60% and 80% among primiparous women [30]. Conversely, participation rates among multiparous women tend to be lower, often below 30–40%. In middle- and low-income countries, access to prenatal education is frequently restricted to urban, more educated populations, with participation rates often falling below 20–30% [32,33]. These disparities reflect differences in healthcare infrastructure, coverage of associated costs, and cultural or social determinants [34,35,36,37,38].

At the European level, there is currently no standardized, harmonized database for collecting data on antenatal education participation. Available data predominantly originate from national surveys and research initiatives. Overall, the trend indicates that Northern European countries, such as Sweden, Norway, Denmark, and the United Kingdom have historically demonstrated high participation rates, often exceeding 80% among first-time mothers. This is largely attributable to their robust public healthcare systems that actively integrate and promote antenatal education services. Conversely, participation rates in Southern and Eastern European countries tend to be lower and exhibit greater heterogeneity [39].

The primary source of these observations is the Euro-Peristat Project [40], a leading initiative for the comparative analysis of perinatal health across Europe. However, the data utilized are over a decade old. The Euro-Peristat report includes a comprehensive set of indicators related to antenatal care, yet reporting consistency varies among countries. Participation in childbirth preparation courses ranges from as high as 90% in Iceland and 86% in Slovenia to as low as 24% in Lithuania. Many countries, such as France with its Enquêtes Nationales Périnatales [41], collect these data periodically; however, Italy has not provided data for this specific indicator within such surveys.

In Italy, childbirth preparation courses promoted by the Ministry of Health, are recommended but not mandatory. Data collection is more accessible at the national level, primarily through the Italian National Institute of Statistics (ISTAT) survey “Pregnancy, Childbirth, and Breastfeeding” [42], which represents the most recent national sample survey on this topic. Nevertheless, this survey is outdated, reporting a participation rate of 58.5% among women who gave birth in 2013 and attended a childbirth preparation course. Participation was notably higher among primiparous women (over 75%) and those with higher educational attainment residing in Northern Italy.

The Ministry of Health and regional authorities periodically publish reports—such as the “Certificate of Birth Assistance” (Certificato di Assistenza al Nascituro, CeDAP)—which contain extensive data on childbirth; however, these reports do not systematically include information on participation in antenatal education. Some regions, including Emilia-Romagna and Tuscany, maintain more detailed monitoring systems that may incorporate this indicator at the local level [43,44,45,46].

Recent literature [47] indicates that participation in childbirth-related educational programs remains significantly associated with educational level, geographical location (higher in the North compared to the South), and socioeconomic status. Maternal age also influences participation, with women over 35 years and primiparous women more likely to attend such courses [48].

Regarding neonatal care practices, the benefits of skin-to-skin contact are well-documented and widely accepted within the scientific community. These benefits encompass nutritional, immunological, growth-related, neurodevelopmental, sleep–wake cycle regulation, and psychosocial advantages [14,15,16,17,18,49,50,51]. Facilitating unrestricted skin-to-skin contact immediately after birth promotes neonatal sleep, a critical component of optimal development, and enhances parent–infant bonding. Implementation should commence as early as possible postpartum.

In the current survey, 2 out of 29 centers do not perform skin-to-skin contact at all. Additionally, in 44.4% of centers, this practice is carried out for less than two hours, though the specific duration is often unspecified, while routine procedures are postponed to some extent in most centers to prioritize skin-to-skin contact. A difference has been observed between Level I and Level II centers, with Level II centers more frequently performing skin-to-skin contact during the first two hours after birth, but it is not statistically significant.

These findings underscore the need for increased support and education regarding the importance of early skin-to-skin contact, particularly within Level I centers.

Immediate and sustained skin-to-skin contact immediately after birth is widely recognized by the WHO and all major scientific societies as a fundamental intervention for optimizing maternal and neonatal health outcomes [6]. Nevertheless, implementation rates vary significantly based on geographical context, delivery mode, and institutional policies. The WHO recommends uninterrupted, early SSC as a universal standard of care [6], emphasizing its role in fostering mother–infant bonding, supporting exclusive breastfeeding, and enhancing neonatal stability [5].

A recent Cochrane review demonstrated that approximately 75% of infants who received early SSC were exclusively breastfed at one month postpartum, compared to 55% among infants who did not experience SSC. Additionally, infants benefiting from early skin-to-skin contact showed improved physiological stability, including more consistent glucose levels, thermoregulation, respiratory patterns, and cardiovascular stability [14].

Despite robust evidence supporting immediate SSC for all newborns, including those born via cesarean section or as stable preterm infants, its implementation remains inconsistent worldwide. This discrepancy contributes to significant disparities both across and within countries. Adoption rates vary considerably depending on national policies, healthcare facility practices, and local cultural factors [52].

The primary global reference is the WHO/UNICEF “Capture the Moment” report [53], which analyzed data from 92 countries across different income levels. On average, only 42% of newborns are placed in skin-to-skin contact with their mothers within the first hour of life. The highest prevalence is observed in East Asia and the Pacific (59%), whereas the lowest is in Eastern and Southern Africa (31%). The report estimates that approximately 78 million newborns annually, about three in five, are separated from their mothers during this critical period. Even in high-income settings, SSC is not universally practiced; for example, CDC data from the Pregnancy Risk Assessment Monitoring System (PRAMS) indicate rates of approximately 60–70% in the United States, with notable disparities influenced by ethnicity, socioeconomic status, and mode of delivery. Routine hospital practices, maternal or neonatal conditions (such as cesarean sections or maternal fatigue), perceptions of the need for neonatal observation, staffing constraints, lack of training, and cultural beliefs are among the key barriers to SSC implementation [54].

A systematic review conducted in 2018 [52] highlighted that worldwide adherence to immediate and sustained SSC remains suboptimal, with reported prevalence rates ranging from as low as 10.2% to as high as 98.9%. In Australia, estimates of SSC prevalence range from 72% to 95% [54]. Conversely, studies from the Western Pacific region report lower rates, with only 20% of Japanese mothers and 10% of mothers in the Philippines practicing SSC [55,56]. In low-income settings, SSC remains markedly underutilized; for example, less than 1% of women in Tanzania adopt SSC [57], and only 9% of women in Ethiopia experience SSC following normal delivery [58,59].

In detail, within this review, the overall prevalence of SSC immediately postpartum ranged from a minimum of 1% in Tanzania [57] to a maximum of 98% in Croatia [35]. Data from the Danish Medical Birth Registry, encompassing 269,597 births, indicate that 96% of women experienced SSC following normal delivery [60]. Despite variations in sample size and context, studies from France and the United Kingdom report similar SSC rates of approximately 64% [61,62], whereas in Switzerland, 95% of mothers experienced SSC within the first hour postpartum [62]. Spanish studies report comparable figures, with SSC observed in 27% and 29% of cases [63,64].

According to official institutional data, the landscape of skin-to-skin contact and early breastfeeding practices across Europe is heterogeneous. Northern European countries generally exhibit higher rates of SSC, often exceeding 90% for vaginal births, whereas Southern and Eastern European nations tend to report lower implementation levels. The Euro-Peristat study [40] indicates that despite some variability in data reporting, countries such as Sweden, Norway, Estonia, and Slovenia demonstrate notably high SSC rates. However, the consistency of data collection remains a challenge.

The WHO/UNICEF Baby-Friendly Hospital Initiative serves as a pivotal driver of change, mandating that certified hospitals demonstrate the practice of continuous, immediate skin-to-skin contact for at least one hour in at least 80% of deliveries. Adoption of this certification, however, varies considerably between countries, influenced by organizational barriers, staffing shortages, and routine clinical practices, as highlighted by the European Foundation for the Care of Newborn Infants [65].

In Italy, a population-based study reported that approximately 80% of women recalled experiencing SSC and initiating breastfeeding within the first hour postpartum at the time of discharge [66]. Nevertheless, Italy lacks a comprehensive national registry; data are primarily derived from survey-based studies and regional reports. The SNLG-ISS guidelines “Cesarean Section: An Appropriate and Informed Choice” (2022) [67] explicitly recommend skin-to-skin contact even following cesarean delivery, provided maternal and neonatal conditions are stable.

The 2015 survey conducted by the Italian Ministry of Health and ISTAT, encompassing approximately 25,000 births, found that 67% of mothers reported having experienced early SSC. Although this figure was below targeted goals, it was considered outdated at the time [41]. The “Nascere in Italia” (“Born in Italy”) survey (2016–2017), coordinated by the Italian National Institute of Health (ISS) and the Center for Child Health [67], revealed substantial regional variability, with SSC rates ranging from 40% to 85%. The study underscored that SSC was less frequently practiced following cesarean sections and in high-volume maternity hospitals.

Recent data from individual hospitals and networks, such as those participating in the UNICEF Baby-Friendly Hospitals initiative, report SSC rates often exceeding 90% for uncomplicated vaginal deliveries. However, the national average remains lower. The Perinatal Health Observatory in Lombardy provides disaggregated data, illustrating significant variability among birth centers. The most recent study from 2024 reports an overall SSC adherence rate of 95.5%. Nonetheless, only 43.8% of participating centers, representing approximately 27% of Italian facilities, predominantly in the North, apply SSC exclusively after vaginal delivery, with 51% implementing SSC regardless of mode of delivery [68]. In southern regions, the practice differs markedly, as exemplified by our institutional experience.

The practice of rooming-in is widely recognized for its positive impact on breastfeeding success [14,18,19] and the development of caregiving skills. In our survey, over 70% of respondents reported implementing rooming-in, which we consider a favorable indicator of adherence to best practices.

However, optimism regarding overall rooming-in rates must be tempered when examining data on 24 h rooming-in, which stands at approximately 27.6%. This relatively low figure may be linked to reports from half of the centers indicating the absence of a caregiver remaining in the room continuously. Maternal–infant separation should be avoided due to its well-documented negative effects on both mother and child [69,70]. When logistical or organizational barriers impede continuous rooming-in, targeted interventions should be pursued through collaborative efforts between hospital administration and relevant health authorities. The objective is to facilitate uninterrupted family–infant proximity around the clock, while also addressing associated risks such as maternal exhaustion. Notably, nine centers require informed consent specifically due to these concerns.

Discharge predominantly occurs within 72 h, emphasizing the critical importance of effective rooming-in practices. Such proximity is essential for hands-on training in neonatal care, particularly for first-time mothers.

The WHO recommends rooming-in as a standard practice [71], and many countries have implemented policies to promote its adoption. Although estimates indicate that the prevalence of rooming-in exceeds 70–80% in numerous developed nations [21], adoption rates vary considerably across different regions.

High adherence is observed in Nordic countries and much of Western Europe, while rates are increasing but not universal in North America, Australia, and parts of Latin America [72]. Conversely, in many regions of Africa, Asia, and the Middle East, the practice remains less common due to cultural norms, infrastructural limitations, or resource constraints. Hospitals accredited by the BFHI typically implement rooming-in as a core requirement [73].

Globally, there is no single percentage that can be universally applied. In high-income countries with robust public health policies, the practice is now standard in approximately 80–95% of physiological births. However, worldwide, the percentage drops considerably, likely below 50%, due to disparities in healthcare systems. Within Europe, rooming-in is generally regarded as a standard practice. Adherence rates are exceptionally high, approaching 100% for physiological births, supported by clear guidelines, comprehensive staff training, and a cultural emphasis on bonding and breastfeeding.

Practices vary within Europe: in Nordic countries (Sweden, Norway, Denmark, Finland), 24 h rooming-in is the absolute norm, with rates near 100% for uncomplicated deliveries, and maternal–infant separation is a rare, justified exception. In Western Europe (France, Germany, the UK, Spain, Benelux), the practice is well established, with rates between 90 and 98%, although logistical challenges may persist in older or overcrowded facilities. Southern and Eastern European countries also demonstrate high and increasing adherence, supported by EU directives and BFHI initiatives, with average rates exceeding 90% [74]. Variations exist between urban centers, where adherence approaches universality, and peripheral regions.

In Italy, rooming-in practices are notably widespread. According to data from the ISS [75], derived from the national surveillance system and the most recent survey conducted between 2021 and 2023, over 95% of maternity units provide 24 h rooming-in. While the majority of Italian hospitals implement continuous rooming-in, occasional temporary interruptions, primarily during overnight hours, are primarily attributable to organizational constraints. This trend is gradually decreasing. Maternal adherence to rooming-in is similarly high, with over 95% of mothers who experience uncomplicated deliveries and have healthy neonates practicing rooming-in. Exceptions are limited to cases involving maternal or neonatal clinical conditions that require intensive care, or less frequently, organizational challenges such as overcrowding.

In 11 centers, the specific type of formula milk is indicated at discharge, with the stipulation that its use is contingent upon medical indications determined by the discharging physician or, subsequently, by the family pediatrician. This practice is subject to debate, as it may be perceived as conflicting with regulations governing the marketing of breast-milk substitutes. However, centers adopting this approach emphasize the importance of medical prescription to reduce the risk of self-prescription or over-the-counter purchasing, practices that may not align with, or could potentially undermine, infant health.

It is essential that any prescription at discharge be accompanied by comprehensive counseling to reinforce that formula feeding should be considered solely in cases of genuine medical necessity. Furthermore, counseling should underscore that the use of breast-milk substitutes can diminish the likelihood of exclusive breastfeeding, which is a critical determinant of health outcomes both in the short and long term.

Recent evidence [76] indicates that the duration and maintenance of exclusive breastfeeding are multifactorially determined by factors such as prenatal care, maternal education, parity, mode of delivery, and immediate skin-to-skin contact. Among these, in-hospital supplementation with infant formula within the first 48 h emerges as a critical and independent barrier to successful breastfeeding initiation and continuation. This underscores the importance of comprehensive public health policies and multidisciplinary interventions aimed at protecting, promoting, and supporting breastfeeding from the immediate postpartum period.

Post-discharge postpartum care for breastfeeding mothers must extend beyond hospital stay, especially in light of trends toward early discharge. Currently, only 41% of facilities offer a brief follow-up appointment with nursing or midwifery staff, while merely 27% provide follow-up with medical personnel, and approximately 21% offer dedicated telephone consultation services for breastfeeding support. These relatively low figures are largely attributable to personnel shortages, a challenge particularly acute in Level II hospitals. Given the critical importance of sustaining exclusive and prolonged breastfeeding for infant health and survival, these gaps represent a vital area for policy improvement. There is a pressing need to bolster support systems, including the integration of dedicated breastfeeding specialists within the national healthcare framework, models successfully implemented in other regions, to enhance post-discharge breastfeeding outcomes.

Although Italy has taken steps with the Hospital Policies on Breastfeeding Project, the accreditation of the Baby-Friendly Hospital Initiative remains a valid and essential pathway, although it has been minimally implemented to date. This survey strongly suggests expanding membership and adherence to this initiative, which currently represents the most effective approach in terms of achieving higher rates of exclusive and prolonged breastfeeding.

The limitations of the study are as follows: the small sample size reduces statistical power and increases the risk of error; consequently, the findings should be considered preliminary and require confirmation in larger cohorts. However, the results provide useful insights into the centers that most require additional training to standardize outcomes across the entire territory. It would be valuable to expand the sample to include more Italian regions; as a transversal (cross-sectional) survey, the study captures data at a single point in time, limiting the ability to infer causality or observe changes over time in breastfeeding practices, but the importance of conducting the survey repeatedly in a serial manner has been underscored, in order to establish it as a monitoring tool essential for continuous improvement. The reliance on self-reported data via online questionnaires may introduce response bias, with centers possibly over-reporting adherence to recommended practices or providing socially desirable responses; however, the physicians responsible for collecting the data received instructions from the Authors to report only corresponding data and to in no way deviate from the actual circumstances to appear more favorable; the survey appears to focus primarily on the existence of practices (e.g., rooming-in, SSC) rather than their actual implementation quality, consistency, or duration, which can vary significantly; the study primarily provides quantitative data, which may overlook contextual factors, staff attitudes, or infrastructural barriers impacting practice implementation, and we are aware of these limitations and have planned a study to evaluate the quality of practices conducted in birth centers; the analysis does not directly assess breastfeeding rates or maternal and neonatal health outcomes, limiting understanding of the actual impact of these practices. The comparison with outcomes is extremely interesting and should translate into improved results, as has been extensively demonstrated over time with robust evidence. However, this aspect is beyond the scope of the study, which implicitly assumes that a more widespread use of practices facilitating the initiation of breastfeeding will lead to better outcomes, both in terms of breastfeeding duration and clinical benefits.

## 5. Conclusions

This survey underscores the critical importance of monitoring adherence to breastfeeding promotion practices within a regional context, situated within broader national and global frameworks. Locally, these findings provide a valuable foundation for enhancing the performance of individual neonatal units in relation to this vital aspect of neonatal health. Furthermore, the survey offers key insights into potential intervention points for aligning clinical practices with well-established, evidence-based guidelines spanning from pregnancy through post-discharge care.

The differences observed between units highlight the necessity for targeted interventions to ensure the consistent implementation of all known strategies for promoting breastfeeding initiation. From a broader perspective, the analysis reveals notable territorial disparities across different facets of neonatal care, reflecting the diverse realities faced by various regions. Many of these challenges can be addressed through policy modifications and practice improvements that do not require substantial financial investment but rather a cultural shift and changes in approach.

Adopting and adapting successful initiatives from other countries could facilitate these improvements, ultimately advancing the goal of achieving universal standards in neonatal care as outlined by the World Health Organization.

## Figures and Tables

**Table 1:** Table of answers to the questionnaire.

**Childbirth Education Classes**			**Skin to Skin Contact**			**Rooming in**		
	**N°**	**%**		**N°**	**%**		**N°**	**%**
	11/29	38%	~2 h	15/29	51.7	Yes	29	100
	Participation		<2 h	12/29	41.3	No	0	0
<30%	10	34.5	No	2/29	6.9	Total	8/29	27.6
30–50%	10	34.5	Checks during the SSC			Written consent		
50–75%	2	6.9	Nurse/midwife	16/20	80	Yes	9/29	31
>75%	4	13.7	Medical	4/20	13.8	Presence of a caregiver		
						Yes	14/29	48.3
**Legend Formulated Milk**			**Obstetric/nursing check-up** **Post-discharge**			**Medical check-up within the first week**		
	N°	%		N°	%		N°	%
Yes	11	39.3	Yes	12	41.4	Yes, all	14/28	50
No	17	60.7	No	17	58.6	Yes, only primiparous	8/28	28.6
						No	6/28	21.4
			**Telephone breastfeeding advice**					
				N°	%			
			Yes	6/27	22.2			
			No	21/27	77.8			

**Table 2:** Procedure delay and type of unit correlation.

Contingency Table			
	Procedure Delay		
Type of Unit	No	Yes	Total
1st level	2	7	9
2nd level	0	4	4
Total	2	11	13

**Table 3:** Procedure delay and type of unit correlation.

Test χ^2^			
	Value	gdl	*p*
χ^2^	1.05	1	0.305
N	13		

**Table 4:** Distribution of adherence to skin-to-skin practice in the first two hours after delivery by unit type.

Contingency Table			
	SSC 2 Hours		
Type of Unit	No	Yes	Total
1st level	9	10	19
2nd level	5	5	10
Total	14	15	29

**Table 5:** Correlation between unit type and adherence to skin-to-skin practice.

Test χ^2^			
	Value	gdl	*p*
χ^2^	0.0182	1	0.893
N	29		

**Table 6:** Contingency table.

Contingency Table			
	Rooming-in		
Type of Unit	Partial	24 h/24 h	Total
1st level	13	6	19
2nd level	7	3	10
Total	20	9	29

**Table 7:** Rooming-in and type of unit correlation.

Test χ^2^			
	Value	gdl	*p*
χ^2^	0.00763	1	0.930
N	29		

**Table 8:** Contingency table. Availability of a post discharge visit dedicated to breastfeeding and type of unit.

Contingency Table				
	Post-Discharge Follow Up for Breastfeeding			
Type of Unit	No	Yes Primiparous	Yes All	Total
1st level	3	4	11	18
2nd level	3	4	3	10
Total	6	8	14	28

**Table 9:** Availability of a post discharge visit dedicated to breastfeeding and type of unit correlation.

Test χ^2^		
Value	gdl	*p*
2.49	2	0.288
28		

## Data Availability

The original contributions presented in this study are included in the article/Supplementary Material. Further inquiries can be directed to the corresponding author.

## Supplementary Materials

The following supporting information can be downloaded at: https://www.mdpi.com/article/10.3390/children13081078/s1, Table S1: The STROBE document—List of items that should be considered in the publication of observational studies.
