# Supplementary material for: Factors Influencing Successful Breastfeeding: A Regional Survey and Comparative Review in European and Global Contexts
Source: Children (Basel). 2026 Aug 14;13(8):1078. doi: 10.3390/children13081078 (PMC13510415; doi:10.3390/children13081078)
Supplement: Supplementary file 1 [file children-13-01078-s001.zip › children-4380943-supplementary.pdf]

| **Table S1.** The STROBE document - List of items that should be considered in the publication of observational studies | | |
|---|---|---|
| | Item number | Recommendation |
| **TITLE and ABSTRACT** | 1 | (a) Indicate the study design in the title or abstract using a commonly used term. Done<br>(b) Provide in the abstract a comprehensive and balanced summary of what was done and what was observed. We think we did. |
| **INTRODUCTION** | | |
| Premises / Rationale | 2 | Explain the scientific background and rationale of the research proposed for publication. We think we have explained this extensively |
| Objectives | 3 | Indicate the specific objectives, including all prespecified assumptions. We have indicated them in several places |
| **METHODS** | | |
| Study design | 4 | Present the fundamental elements of the study design in the initial part of the work |
| Context | 5 | Describe the relevant context, locations and dates, including recruitment, exposure, follow-up and data collection periods. We have indicated them. |
| Participants | 6 | *Cross-sectional study* – State eligibility criteria, sources of participants, and methods of participant selection.<br>We believe we have indicated this correctly |
| Variables | 7 | Clearly define all outcomes, exposures, predictors, possible confounders, and effect modifyers. Indicate diagnostic criteria, if applicable. We have chosen to study the factors most significantly impacting, according to the literature and evidence-based data, the success of breastfeeding, and which could be reported by each center while minimizing the risk of bias. |
| Data sources / survey | 8* | For each variable of interest, indicate the sources of the data and explain in detail the evaluation methods (survey).<br>This is a questionnaire that has been detailed.<br>Explain the comparability of assessment methods if there is more than one group. Subgroups emerged in the analysis and the comparison was specified. |
| Systematic errors (bias) | 9 | Describe all efforts to consider possible sources of bias. They have been specified in the paragraph of limits with mitigation measures. |
| Size of the study | 10 | Explain how the size of the study was reached. It was specified that the study considered eligible each birth center present in the analyzed territory which includes two regions (Puglia and Basilicata) |
| Quantitative variables | 11 | Explain how quantitative variables were handled in the analyses. If applicable, describe which groupings were chosen and why. Groups are derived from the responses to the questions, and the numbers that define them in absolute terms, percentages, and distribution are clearly specified in the results section. |

| | | |
|---|---|---|
| Statistical methods | 12 | 1. Describe all statistical methods, including those used to control for confounding factors<br>2. Describe the methods used to examine subgroups and interactions<br>3. Explain how missing data was handled<br>4. *Cohort Study* - If applicable, explain how the loss was treated over the course of follow-up<br>*Case-control study* - If applicable, explain how case matching with controls was treated<br>*Cross-sectional study* - If applicable, describe analytical methods taking into account the sampling strategy<br>Statistical methods have been specified |
| **RESULTS** | | |
| Participants | 13* | Report the number of individuals at each stage of the study - e.g., number of potentially eligible individuals, screened for eligibility, confirmed as eligible, included in the study, who completed follow-up, and who were analyzed<br>Indicate the reasons for not participating in each stadium<br>We do not know the reasons for the lack of responses but the non-response rate was very low<br>(a) Consider using a flowchart. Not indicated |
| Descriptive data | 14* | 1. Indicate the characteristics of the study participants (e.g., demographic, clinical, social) and give information on exposures and potential confounders<br>Indicate the number of participants with missing data for each variable of interest. It has been specified.<br>(a) *Cohort Study* – Summarize the duration of follow-up (e.g ., average and total) |
| Outcome data | 15* | Cross-sectional study - Report the number of outcome events or summary measures. The results section is included and has been improved in the latest version. |
| Main results | 16 | 1. Provide unadjusted estimates and, if applicable, adjusted estimates for confounding factors and their accuracy (e.g., 95% confidence interval). Clarify which confounding factors the adjustment was made for and why they were included<br>2. Report category boundaries when categorizing continuous variables<br>3. If relevant, consider translating relative risk estimates into absolute risk over a significant period of time<br><br>Statistical analysis was performed utilizing Jamovi statistical software (Version 2.3; Jamovi project, 2022). Descriptive statistics were applied to summarize the data; specifically, categorical variables were expressed as absolute frequencies and corre-sponding percentages.<br>To explore potential relationships between variables and identify statistically significant correlations, Pearson's or Spearman's correlation coefficients were computed based on the underlying data distribution (parametric or non-parametric, respective-ly). Furthermore, to evaluate associations between qualitative (categorical) variables, contingency tables were generated. These associations were subsequently analyzed using the Chi-square test or Fisher's exact test, as appropriate, depending on expected cell counts |
| Other analyses | 17 | Report other analyses performed – e.g., subgroup-pi and interaction analyses and sensitivity analyses |
| **DISCUSSIONE** | | |
| Main results | 18 | Summarize the main findings in relation to the objectives of the study. In this work, in addition to providing an interpretation of the results based on the objectives, a review of the available national and supranational data |

| | | |
|---|---|---|
| | | from the literature on the various points of analysis was conducted. |
| Limitations | 19 | Discutere i limiti dello studio, tenendo conto delle eventuali fonti di errori sistematici (bias) o imprecisioni.<br>Discutere sia la direzione sia le dimensioni di tutti i potenziali errori sistematici (bias) |
| Interpretation | 20 | Provide a prudent overall interpretation of the results, taking into account the objectives, limitations, multiplicity of analyses, results obtained in similar studies and other relevant evidence. See the answer to point 18 |
| Generalizability | 21 | Discuss the generalizability (external validity) of the study results . This aspect has been explored extensively |
